# Novel Fluorescent Mitochondria-Targeted Probe MitoCLox Reports Lipid Peroxidation in Response to Oxidative Stress *In Vivo*

**DOI:** 10.1155/2020/3631272

**Published:** 2020-02-10

**Authors:** Konstantin G. Lyamzaev, Alisa A. Panteleeva, Anna A. Karpukhina, Ivan I. Galkin, Ekatherina N. Popova, Olga Yu. Pletjushkina, Bettina Rieger, Karin B. Busch, Armen Y. Mulkidjanian, Boris V. Chernyak

**Affiliations:** ^1^Belozersky Institute of Physico-Chemical Biology, Lomonosov Moscow State University, Moscow 119992, Russia; ^2^Department of Bioengineering and Bioinformatics, Lomonosov Moscow State University, Moscow 119992, Russia; ^3^Institute of Molecular Cell Biology, Department of Biology, University of Muenster, D-48149 Muenster, Germany; ^4^Mitochondrial Dynamics Group, Department of Biology, Osnabrueck University, D-49069 Osnabrueck, Germany; ^5^Department of Physics, Osnabrueck University, D-49069 Osnabrueck, Germany

## Abstract

A new mitochondria-targeted probe MitoCLox was designed as a starting compound for a series of probes sensitive to cardiolipin (CL) peroxidation. Fluorescence microscopy reported selective accumulation of MitoCLox in mitochondria of diverse living cell cultures and its oxidation under stress conditions, particularly those known to cause a selective cardiolipin oxidation. Ratiometric fluorescence measurements using flow cytometry showed a remarkable dependence of the MitoCLox dynamic range on the oxidation of the sample. Specifically, MitoCLox oxidation was induced by low doses of hydrogen peroxide or organic hydroperoxide. The mitochondria-targeted antioxidant 10-(6′-plastoquinonyl)decyltriphenyl-phosphonium (SkQ1), which was shown earlier to selectively protect cardiolipin from oxidation, prevented hydrogen peroxide-induced MitoCLox oxidation in the cells. Concurrent tracing of MitoCLox oxidation and membrane potential changes in response to hydrogen peroxide addition showed that the oxidation of MitoCLox started without a delay and was complete during the first hour, whereas the membrane potential started to decay after 40 minutes of incubation. Hence, MitoCLox could be used for splitting the cell response to oxidative stress into separate steps. Application of MitoCLox revealed heterogeneity of the mitochondrial population; in living endothelial cells, a fraction of small, rounded mitochondria with an increased level of lipid peroxidation were detected near the nucleus. In addition, the MitoCLox staining revealed a specific fraction of cells with an increased level of oxidized lipids also in the culture of human myoblasts. The fraction of such cells increased in high-density cultures. These specific conditions correspond to the initiation of spontaneous myogenesis *in vitro*, which indicates that oxidation may precede the onset of myogenic differentiation. These data point to a possible participation of oxidized CL in cell signalling and differentiation.

## 1. Introduction

Cardiolipin (CL) is a unique diphosphatidylglycerol phospholipid with four acyl chains. In eukaryotic cells, it is exclusively located in the inner mitochondrial membrane where it constitutes about 18% of phospholipids. CL supports the functional activity of mitochondria by shaping the membrane curvature and defining the crista morphology [1], stabilizing respiratory supercomplexes [2–5], mediating proton transfer to energy-converting enzymes [6–8], and preventing proton leakage [9]. Almost all membrane energy-converting enzymes contain tightly bound CL molecules as important structural components [10–12].

In intact mitochondria, CL is located exclusively in the inner membrane, but various mitochondria-damaging agents induce translocation of CL to the outer mitochondrial membrane [13]. It was found that nucleoside diphosphate kinase D (NDPK-D) binds CL in the intermembrane space and facilitates its redistribution [13, 14]. The externalized CL can interact with the dynamin-related GTPase Drp1 and stimulate its oligomerization, which is critical for the fission of mitochondria [15]. Interestingly, the other dynamin-related GTPase OPA1, which stimulates the inner membrane fusion, also interacts with CL [13, 16]. In addition, CL molecules exposed at the surface of fragmented mitochondria can be recognized by receptors of autophagosomes including LC3, which induces the engulfment of damaged organelles (mitophagy) followed by their digestion in lysosomes [17]. The other important partners of externalized CL are inflammasome NLRP3 [18] and caspase-1 [18]. It was suggested that independent interactions of the NLRP3 and caspase-1 with CL at the outer mitochondrial membrane contribute to the proinflammatory activation of macrophages [19]. These findings indicate that CL is an important player in the regulation of mitochondrial dynamics and in the mechanisms of quality control.

The content of unsaturated fatty acids in CL is significantly higher than that in other mitochondrial phospholipids. In combination with the proximity to respiratory enzymes, which are potential sources of reactive oxygen species (ROS), the high content of unsaturated fatty acids makes CL especially sensitive to oxidation. It was found that a small respiratory protein cytochrome *c*, after its binding to oxidized CL at the outer surface of the inner mitochondrial membrane, can catalyze CL peroxidation, which induces the cytochrome *c* release from mitochondria and apoptosis [20, 21]. Oxidation and age-dependent loss of CL were suggested to contribute to age-related cardiac [22] and neurodegenerative [23] diseases, as well as to diabetes [24]. It was suggested that the protective effects of mitochondria-targeted peptide SS-20 owe to its binding to CL and preventing cytochrome *c*-dependent CL peroxidation [25]. Imidazole-substituted analogs of fatty acids that were conjugated with TPP^+^ inhibited cytochrome *c*-dependent CL peroxidation and protected mouse embryonic cells exposed to ionizing irradiation [26]. As argued elsewhere, the protective and antiaging effects of mitochondria-targeted antioxidants with various cationic maieties could be due to the specific protection of CL against peroxidation [27, 28].

**Figure 1:**
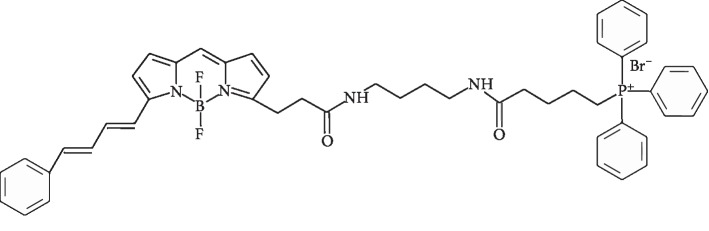
Chemical structure of MitoCLox.

Elsewhere, we have described MitoCLox, a new mitochondria-targeted fluorescence probe for tracing cardiolipin (CL) oxidation [29]. In MitoCLox, similar to the previously introduced MitoPerOx [30], a BODIPY (581/591) fluorophore is linked with a triphenylphosphonium cation (TPP^+^). However, the linker in MitoCLox is longer than the linker of MitoPerOx and contains not one but two peptide bonds (see Figure 1). The flexible linker was chosen as a mimic of the SS-20 peptide from Ref. [25]; this long linker has a capacity to accommodate additional positively charged moieties.

It was shown that MitoCLox can report CL oxidation in liposomes but did not react with organic hydroperoxides even in the presence of ferric ions [29]. Based on results of molecular dynamic simulations, it was suggested that MitoCLox and its derivatives, owing to the positive charge(s), could be selectively sensitive to oxidation of cardiolipin, the dominant negatively charged phospholipid in the inner mitochondrial membrane [29].

Here, we have used MitoCLox for tracing peroxidation of mitochondrial lipids in living cells. MitoCLox selectively accumulated in their mitochondria and reported lipid peroxidation induced by exogenous prooxidants or by internal redox changes.

## 2. Materials and Methods

### 2.1. Chemicals

MitoCLox was synthesized as described in [29]. SkQ1 was synthesized, as described in [31]. Other reagents were from Sigma-Aldrich (USA).

### 2.2. Cell Cultures

Human carcinoma cell line RKO (ATCC CRL-2577), human fetal lung fibroblasts MRC5 transformed with SV-40 (MRC5 SV2, EcACC Cat. No. 84100401), and human endothelial cell line EA.hy926 (ATCC CRL-2922) were cultured in DMEM medium (Dulbecco's modified Eagle's medium) (Gibco, USA) supplemented with 2 mM glutamine and 10% fetal bovine serum (FBS) (HyClone, USA) and 100 U/ml streptomycin and 100 U/ml penicillin (all from Gibco, CA). HeLa cells were cultured in minimal essential medium with Earle's salts (MEM, PAA Lab GmbH, E15–888) with 5.6 mM glucose, 2 mM stable glutamine, and sodium bicarbonate, supplemented with 10% FBS (Biochrom AG), 1% MEM nonessential amino acids (Biochrom AG), and 1% 4−(2−hydroxyethyl)piperazine-1-ethanesulfonic acid (HEPES, PAA Lab GmbH). Immortalized human myoblast MB135 were cultured in mixture of DMEM and 199 medium (4 : 1) supplemented with 15% FBS (HyClone, USA), basic fibroblast growth factor FGF-2 (10 ng/ml PanEco, Russia), and 0.1 *μ*Μ dexamethasone.

### 2.3. Microscopy

MRC5-SV40 fibroblasts and EA.hy926 endothelial cells were grown on glass coverslips placed in 6-well cell culture plates at 200,000 cells per well and analyzed using an Axiovert microscope (Carl Zeiss). For analysis of mitochondrial membrane potential, Ea.hy926 cells were incubated with TMRM (100 *μ*M, 30 min) and with MitoTracker Green (250 nM, 30 min). For detection of mitochondrial lipid peroxidation, MitoCLox (200 nM) was added for 2 h. Myoblast MB135 were seeded in 35 mm dishes with glass bottom (SPL) for confocal microscopy at initial density from 0.5 to 6 × 10^5^ cells/dish and cultured for 4 days. Then, MitoCLox (200 nM) was added for 5 h, and cells were analyzed using a Nikon Eclipse Ti (Nikon) confocal microscope with excitation at 488 and 562 nm.

Fluorescence imaging of HeLa cells was carried out with a confocal laser scanning microscope (Leica TCS SP8 SMD) equipped with a 63x water objective (water, HCPL Apo 63x/1.2 W CS2) and two spectral detectors, an Argon and a tunable white light laser. Measurements were performed at 37°C. HeLa cells were incubated with 25 *μ*M menadione or with 100 *μ*M *tert*-butyl hydroperoxide (tBOOH) for 1 h, and 200 nM MitoCLox was added for the indicated time. In the green channel, fluorescence was excited with the 448 nm wavelength of an argon ion laser, and emission was collected in the range of 500–560 nm. In the red channel, fluorescence was excited with the 559 nm laser wavelength of the white light laser, and emission was recorded in the range of 580–630 nm.

### 2.4. Image Processing

The fluorescence intensity of HeLa cells stained with MitoCLox was analyzed with ImageJ (MacBiophotonics). To exclude the background intensity, the Otsu mask was used as a mask for mitochondria and the background was set to NaN. Ratios were determined from mean grey values for each channel and represent basically the mitochondrial network of a cell.

### 2.5. Flow Cytometry

MRC5-SV40 cells were incubated with MitoCLox (100-200 nM) for 1 h before addition of H_2_O_2_ or cumene hydroperoxide. SkQ1 was added for 24 h before stimulation of oxidative stress. To measure the mitochondrial membrane potential (MMP), the cells were stained with 100 nM TMRM for 15 min.

Myoblast MB135 were seeded in 6-well cell culture plates (9.6 cm^2^ surface area per well) at initial density 0.5, 1, 2, 4, and 6 (×10^5^ cells/well) and cultured for 4 days. Then, 200 nM MitoCLox was added for 5 h, and cells were analyzed.

Flow cytometry analyses were performed using a Beckman Coulter FC 500, equipped by a single blue (488 nm) laser or BDFACSAria III with 5 lasers (375 nm, 405 nm, 488 nm, 561 nm, and 633 nm). For ratiometric analysis, the Flowing software 2.4 (Cell Imaging Core, Turku Centre for Biotechnology) was used.

### 2.6. Statistics

Data analysis is presented as the mean ± standard deviation (SD). Comparisons were analyzed by one-way ANOVA. The significance was analyzed with Prism 7.0 software (GraphPad, USA); a *p* value < 0.05 was considered to be statistically significant. Data analysis of MitoCLox-stained HeLa cells was performed using Origin™ (OriginLab Cooperation, Northampton, MA). The data are presented as means in box-and-whisker plots, with boxes representing the 25th to 75th percentiles. In order to determine differences between treatment groups, analysis of variance (ANOVA) for a single factor (One-way) was performed with the post hoc Scheffe test. Differences were considered to be statistically significant if *p* < 0.05.

## 3. Results

### 3.1. Interaction of MitoCLox with Mitochondria of Fibroblasts

**Figure 2:**
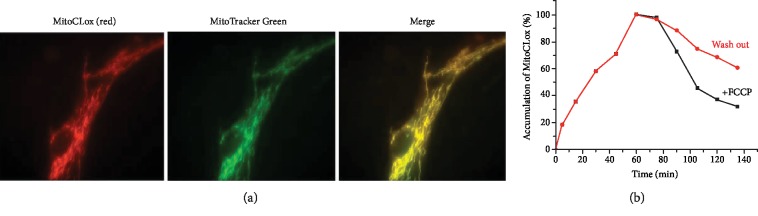
Accumulation of MitoCLox in MRC5-SV40 cells. (a) Cells were incubated with 200 nM of MitoCLox for 60 min; then, 50 nM MitoTracker Green was added for 15 min. Cells were analyzed with an Axiovert microscope (Carl Zeiss). Bar, 10 *μ*m. (b) Accumulation of MitoCLox (200 nM) as measured by FACS in FL2 channel. After 60 min, the medium was changed to the same but without MitoCLox. FCCP (10 *μ*M) was added after change of the medium where indicated.

To assess the ability of MitoCLox to accumulate in the cell mitochondria of human MRC5-SV40 fibroblasts, we used fluorescence microscopy with the red filter corresponding to the fluorescence of the reduced probe. We observed MitoCLox accumulation in fibroblasts and its colocalization with the mitochondria-specific dye MitoTracker Green (Figure 2(a)). The green fluorescence of MitoCLox was not significant since the dye was reduced and did not affect the colocalization analysis.

The dynamics of dye accumulation and the rate of its release from the cells were evaluated using flow cytometry. Maximal accumulation of MitoCLox in the cells was reached in 45-60 minutes (Figure 2(b)). After removal of MitoCLox from the medium, the fluorescence of the cells slowly decreased and reached 50% of the maximum in approximately 1 h. The addition of a membrane depolarizing agent FCCP during probe removal significantly accelerated the release of MitoCLox from the cells (Figure 2(b)), which indicates the dependence of its accumulation on the mitochondrial membrane potential. When FCCP was added before MitoCLox, the fluorescent dye was diffusively distributed all over the cytosol (not shown).

**Figure 3:**
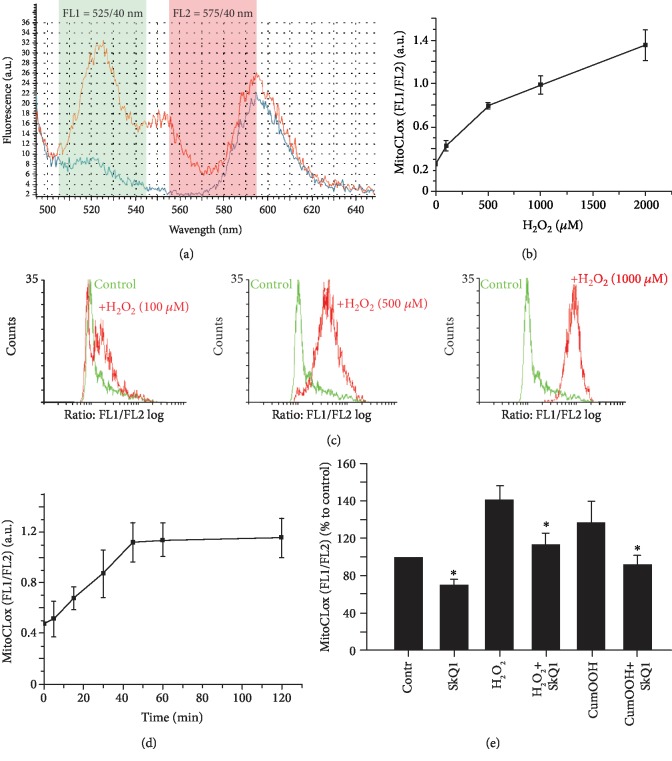
Measurements of MitoCLox oxidation in living cells by flow cytometry. MRC5-SV40 cells were incubated with MitoCLox (200 nM) for 1 h before the addition of peroxides. (a) Fluorescence spectra of reduced MitoCLox (blue line) and MitoCLox oxidized by 500 *μ*M H_2_O_2_ (red line) at 488 nm excitation. The green area is the part of the spectrum detected by the standard bandpass filter 525/40 nm (FL1); the red area is the part of the spectrum detected by filter 575/40 nm (FL2). (b) The ratiometric measurements (FL1/FL2) of MitoCLox oxidation induced by 1 h incubation with H_2_O_2_. (c) An example of typical histograms obtained in experiments where the cells were treated with H_2_O_2_ and MitoCLox oxidation was measured as the FL1/FL2 ratio. (d) The time dependence of the MitoCLox oxidation in response to the addition of H_2_O_2_ (500 *μ*M). (e) Incubation of the cells with mitochondria-targeted antioxidant SkQ1 (20 nM, 2 h) protects the cells from oxidative stress induced by H_2_O_2_ (500 *μ*M) or 25 *μ*M cumene hydroperoxide (CumOOH). All experiments were triplicated, and each bar represents the mean ± SD (^∗^*p* < 0.05 vs. the control or treated cells without SkQ1).

Oxidation of MitoCLox was analyzed in the same fibroblast cells by minimal flow cytometry setup using a single blue (488 nm) laser and standard bandpass filters 525 ± 40 nm (FL1) to record the emission peak of oxidized MitoCLox versus the peak at 575 ± 40 nm (FL2). Although these settings did not perfectly fit the peaks of BODIPY581/591 fluorescence (Figure 3(a)), the changes in the FL1 signal increased 11-fold after the addition of 500 *μ*M H_2_O_2_ to living cells. The FL2 signal also raised slightly, which was mostly due to the contribution from the long wavelength shoulder in the fluorescence spectra of the oxidized form (Figure 3). As a result, the oxidation of MitoCLox resulted in a 5-6-fold increase of the FL1/FL2 emission signal ratio. In an attempt to further improve the sensitivity, we applied the flow cytometry setup with two lasers, namely, the blue laser (488 nm) and green laser (561 nm) and bandpass filters of 530 ± 30 nm (FL1) and 582 ± 15 nm (FL2). These settings fit better the parameters of BODIPY581/591 fluorescence, but the dynamic range of the FL1/FL2 signal for MitoCLox was not significantly better than that in the minimal setup (not shown), so flow cytometry with a single blue laser was used in the further experiments.

The analysis of H_2_O_2_-induced oxidative stress demonstrated that the MitoCLox oxidation occurred only in a fraction of the cell population at low levels of H_2_O_2_. At higher doses of H_2_O_2_, almost normal distribution of oxidized MitoCLox was observed (Figure 3(c)). Kinetics of MitoCLox oxidation induced by 500 *μ*M H_2_O_2_ reached saturation at approximately 60 min (Figure 3(d)). Cumene hydroperoxide (CumOOH) induced similar responses of MitoCLox but at lower doses (not shown). Mitochondrially targeted antioxidant SkQ1 (10-(6′-plastoquinonyl)decyltriphenyl-phosphonium [27]) inhibited oxidation of MitoCLox induced by H_2_O_2_ or by CumOOH (Figure 3(e)).

### 3.2. Measurements of Lipid Peroxidation in Mitochondria of HeLa Cells

**Figure 4:**
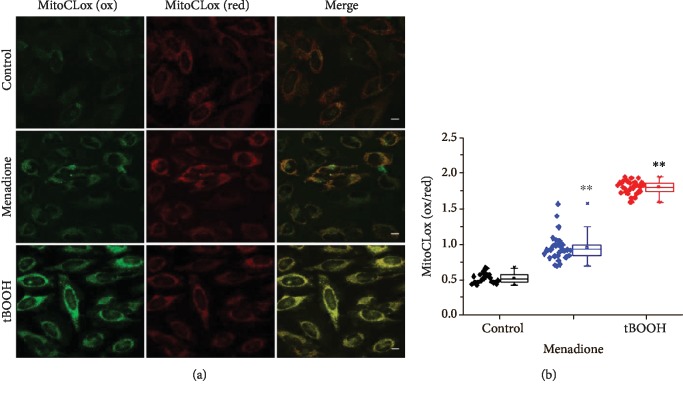
MitoCLox reports CL oxidation in HeLa cells in response to prooxidizing agents. (a) Fluorescence images in the green (*λ*_em_ = 500‐560 nm) and red (*λ*_em_ = 580‐630 nm) emission channels. HeLa cells were incubated with oxidants menadione (25 *μ*M) or *tert*-butyl hydroperoxide (tBOOH, 100 *μ*M) for 1 h, and 200 nM MitoCLox was added for 30 min. Bar, 10 *μ*m. (b) Quantitative ratiometric analysis of MitoCLox response to different prooxidants. SD, ^∗∗^*p* < 0.05 vs. the control.

HeLa cells were pretreated with such oxidizing compounds as menadione (25 *μ*M) and *tert*-butyl hydroperoxide (tBOOH; 100 *μ*M), respectively, for 1 h. In the cells treated with these oxidants, mitochondria were then stained with MitoCLox (200 nM) for 30 min, and the fluorescence in two channels (*λ*_exc1_ = 448 nm/*λ*_em_ = 500‐560 nm; *λ*_exc2_ = 559/*λ*_em_ = 580‐630 nm) was recorded by confocal microscopy (Figure 4(a)). The respective fluorescence images demonstrated significant increase of fluorescence in the green channel and almost no changes in the red channel (Figure 4(a)). Calculations of the green/red fluorescence ratio showed that the increase in the fluorescence ratio (emission ratio *λ*_em_ = 500‐560 nm/*λ*_em_ = 580‐630 nm) after menadione and tBOOH treatment was significant (Figure 4(b)), indicating oxidation of MitoCLox.

### 3.3. Application of MitoCLox for Tracing Separate Steps in Cell Reaction to Oxidative Stress

**Figure 5:**
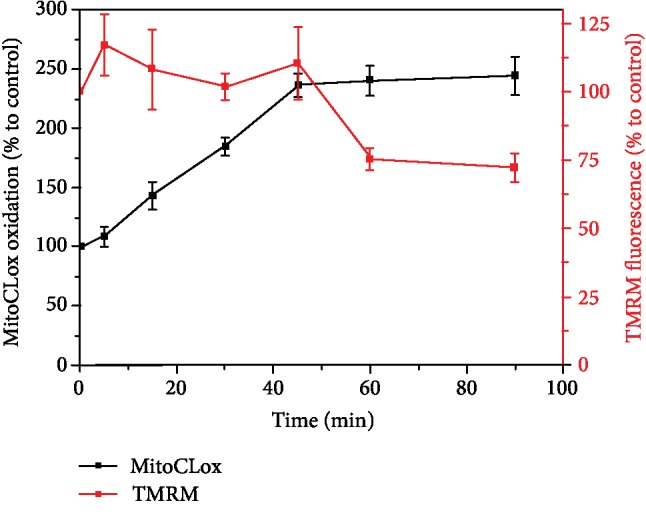
The relationship between lipid peroxidation, as measured by MitoCLox, and decrease in mitochondrial membrane potential under oxidative stress induced by H_2_O_2_. MRC5-SV40 cells were incubated with MitoCLox (200 nM) for 1 h before addition of H_2_O_2_ (0.5 mM). TMRM was added for 15 min before measurements to estimate the membrane potential. Flow cytometry analyses were performed using a Beckman Coulter FC 500. All experiments were triplicated, and each bar represents the mean ± SD.

Oxidative stress is known to cause both mitochondrial lipid peroxidation and a decrease in the mitochondrial membrane potential (MMP) [9, 24]. To analyze the time pattern of lipid peroxidation and MMP decrease under oxidative stress, we measured MitoCLox oxidation concurrently with the potential-dependent accumulation of tetramethylrhodamine (TMRM) in MRC5-SV40 cells (Figure 5). In cells treated with 0.5 mM hydrogen peroxide, oxidation of MitoCLox started without a delay and was almost complete during the first hour (black squares in Figure 5). A notable drop in MMP could be observed after 40 minutes of incubation (red squares in Figure 5).

### 3.4. Heterogeneity of MitoCLox Oxidation in a Single Cell

**Figure 6:**
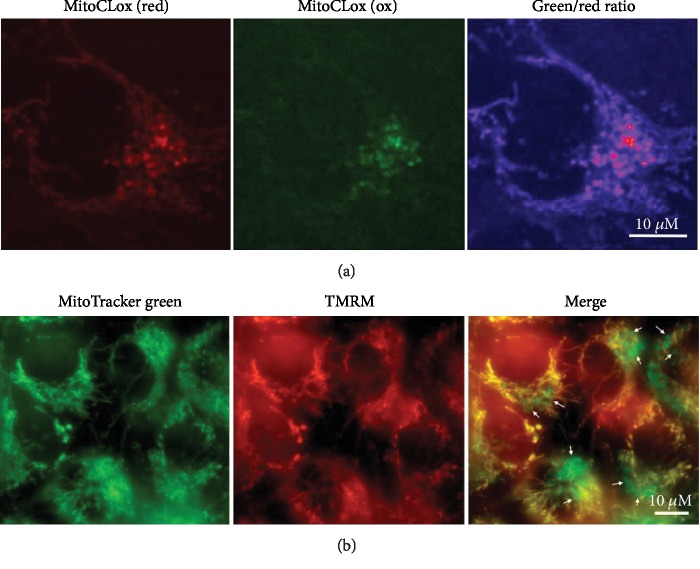
Analysis of mitochondrial heterogeneity in endothelial Ea.hy926 cells. (a) Cells were incubated with MitoCLox (200 nM; 2 h). (b) Cells were incubated with TMRM (100 nM; 30 min) and with MitoTracker Green (250 nМ; 30 min). The arrows indicate the area where mitochondria with decreased membrane potential are located.

Staining of an endothelial cell culture with MitoCLox revealed a fraction of mitochondria with an increased level of lipid peroxidation (Figure 6(a)). These mitochondria were small, rounded, and located near the nucleus.

In Figure 6(b), we show for comparison a similar example of mitochondrial heterogeneity in endothelial cells that we observed earlier [32, 33]. Using a combination of MitoTracker Green that stained mitochondria independently of MMP and methyl ester of TMRM that accumulated only in mitochondria with high MMP, we revealed a fraction of depolarized mitochondria located near the cell nucleus. These perinuclear mitochondria were smaller and more rounded than the mitochondria with higher potential in the same cell. A comparison of images in Figures 6(a) and 6(b) indicates similarities in heterogeneity patterns for the MitoCLox oxidation and MMP in the mitochondrial population.

### 3.5. Application of MitoCLox in Myoblast Cell Culture

We have applied MitoCLox to analyze mitochondrial lipid peroxidation in the culture of human myoblast MB135.

**Figure 7:**
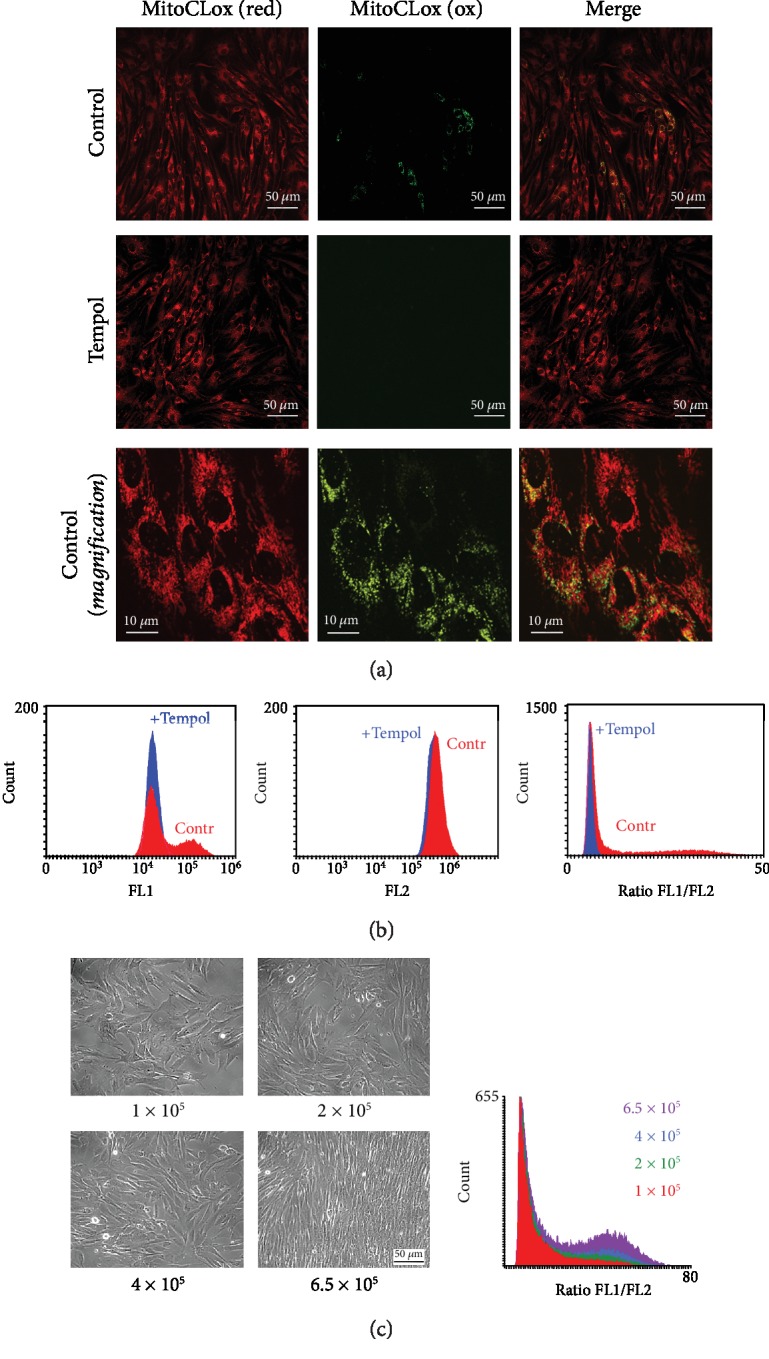
Analysis of MitoCLox oxidation in the culture of myoblast MB135. (a) Myoblasts were seeded in 35 mm dishes at an initial density of 2 × 10^5^ cells/well and cultured for 4 days. Tempol (0.1 mM) was added at the beginning of culturing. Then, 200 nM MitoCLox was added for 5 h, and cells were analyzed using a Nikon Eclipse Ti (Nikon) confocal microscope with excitation at 488 and at 562 nm. Higher magnification was used to reveal mitochondrial morphology. (b) Myoblasts were seeded and cultured as in (a). Then, MitoCLox (200 nM) was added for 5 h, and cells were analyzed using the FC 500 flow cytometer in green (FL1) and red (FL2) channels. (c) Myoblasts were seeded in 6-well cell culture plates (9.6 cm^2^ surface area per well) at initial density 1–6 × 10^5^ cells/well and cultured for 4 days. Then, 200 nM MitoCLox was added for 5 h, and cells were analyzed as in (b). The cell fraction with oxidized MitoCLox was measured. Phase-contrast images of the final cultures seeded at different densities are shown.

Mitochondrial oxidative status is critical for various processes in muscle, including myogenic differentiation (myogenesis)—the process of formation of muscle fibers during embryonic development and muscle regeneration. Myogenesis is accompanied by dramatic reorganization of mitochondrial reticulum through mitophagy and mitochondria biogenesis [34, 35] the processes that critically depend on mitoROS [36]. Thus, analysis of CL peroxidation in myoblast cell cultures could be a valuable tool for studies of myogenesis. We found that a specific fraction of cells exhibited a significant oxidation of MitoCLox in the otherwise homogenous culture of human myoblast MB135. Fluorescence microscopy demonstrated that cells with oxidized MitoCLox formed compact islets in the cell monolayer (Figure 7(a)). The antioxidant Tempol prevented the MitoCLox oxidation. Flow cytometry made it possible to quantify the size of this fraction (Figure 7(b)). The cells with oxidized MitoCLox got accumulated in the cultures that were seeded at an initial density of 1‐6 × 10^5^ cells/well and reached a high density (80-100% confluence) in 4 days of culturing. If the initial seeding density was too low for the cells to become close to confluency after 4 days (0.5 × 10^5^ cells/well), no cells with oxidized MitoCLox were detected. The fraction of the cells with oxidized MitoCLox increased proportionally to the increase of initial seeding density (Figure 7(c)). If the cells were seeded at high density of 6 × 10^5^ cells/well, but cultured only for 24 h instead of 4 days prior to MitoCLox addition, the population of the cells with oxidized MitoCLox was not observed (data not shown). These data allow us to exclude a possible preexistent (either genetic or epigenetic) heterogeneity of the cell culture.

We have observed that mitochondrial fragmentation was significantly more pronounced in the cells with oxidized MitoCLox (Figure 7(a)) in agreement with the key role of mitochondrial ROS in mitochondrial fragmentation [36].

## 4. Discussion

Here, we tested a new mitochondria-targeted LPO fluorescent probe MitoCLox with different cell cultures and under conditions leading to oxidative stress. In all tested cases, MitoCLox was reliable in reporting LPO. The data obtained are fully consistent with the behavior of MitoCLox in the model liposome system [29].

Specifically, MitoCLox accumulated in the cells in a MMP-dependent way and changed its fluorescence in response to addition of H_2_O_2_, menadione, and tBOOH (Figures 2–5). The oxidation of MitoCLox could be prevented by a mitochondria-targeted antioxidant SkQ1 (Figure 3), as well as an antioxidant Tempol (Figure 7), which is in a good agreement with high efficiency of these antioxidants in protection of the mitochondrial structure and functions in various cellular models of oxidative stress [27, 37] and their protective action *in vivo* [38, 39].

It is noteworthy that the response of MitoCLox, as measured by flow cytometry (Figure 3), was more pronounced than that in microscopy experiments (Figures 2 and 4–7), which renders MitoCLox as a suitable marker of lipid oxidation for flow cytometry measurements.

Oxidation of CL increases the proton leak through the inner mitochondrial membrane and causes a drop in MMP [9, 24]. As suggested by Korshunov and colleagues, the drop in MMP protects cells from oxidative damage by suppressing the generation of ROS [40]. Another mechanism of attenuating oxidative damage is the MMP-dependent fragmentation of the mitochondrial network, which allows to separate damaged mitochondria from integer ones and eliminate the former [9, 41]. Since oxidation of CL is also known to serve as a trigger for assembly of apoptosome [42, 43], it appears that the oxidation of CL triggers both pro- and antiapoptotic reactions. As a result, the fate of the cell appears to be determined by the balance between pro- and antiapoptotic reactions triggered by the same event of CL oxidation [9]. For understanding this interplay, it is needed to follow both the CL oxidation and changes in MMP *in vivo*. Figure 5 shows that the drop in MMP follows the oxidation of MitoCLox with a certain lag. Although the reason of the lag deserves further investigation, it seems tempting to speculate that cells could maintain their MMP for tens of minutes by hydrolyzing their ATP stock. Concentration of ATP should not decrease rapidly in highly glycolytic cells that we used, so the expected increase in membrane proton leakage could be initially counteracted by MMP generation by ATPase (and other proton pumps).

Earlier, it was shown that oxidative stress initially results in an exclusive oxidation of CL molecules [44–46]. Therefore, the fast PLO, as reported by MitoCLox in response to oxidative stress in diverse systems (Figures 2–5), was, most likely, due to the predominant oxidation of CL.

The observation of small perinuclear mitochondria with a low MMP (Figure 6) could be related to the earlier report on mitochondrial heterogeneity in endothelial cells [32, 33]. The data in Figures 6(a) and 6(b) indicates the existence of a specific small subpopulation of mitochondria with a reduced MMP and oxidized CL in endothelial cells. Earlier, similar results were reported by Kuznetsov and Margreiter who observed small round perinuclear mitochondria with decreased MMP in HL-1 cardiac muscle cells [47, 48]. It is tempting to speculate that peroxidation of mitochondrial lipids (primarily CL) could be responsible for the decrease in the MMP in the fraction of the mitochondrial population shown by arrows in Figure 6. Probably, these small mitochondria serve as sensors of cellular homeostasis. It is known that oxidative phosphorylation is not a significant source of ATP in endothelial cells [48] so that a small fraction of depolarized mitochondria would not significantly affect the energy balance of these cells. Our observations indicate that CL peroxidation in a fraction of mitochondrial population could be a reason for functional and structural heterogeneity of mitochondria in a single cell.

Application of MitoCLox allowed us to detect a specific fraction of cells with a high level of mitochondrial lipid peroxidation in the culture of human myoblast MB135. The size of this cell fraction increased with an increase in cell density with increasing culturing time. These specific conditions correlate with increased probability of spontaneous myogenesis *in vitro* [49]. At high cell density, cell-cell contacts initiate contact inhibition of the cell cycle progression, cell fusion, and expression of the key players of myogenesis such as MyoD and myogenin [50]. Moreover, myoblasts were shown to secrete their own extracellular matrix glycoproteins that facilitate myogenic differentiation [51]. It is tempting to speculate that a subpopulation of myoblasts with oxidized mitochondrial CL includes the myoblasts that are committed for differentiation.

Fragmentation of the mitochondrial network and mitophagy are required for mitochondrial biogenesis and myogenic differentiation [34, 35]. Deregulation of these processes contributes to various types of genetic muscular dystrophy and in age-associated sarcopenia [36]. Cardiolipin is deeply involved in the regulation of mitochondria dynamics since it interacts both with profission (Drp1) [15] and with profusion (Opa1) [13] dynamin-related GTPases. Furthermore, externalization of CL to the outer mitochondrial membrane could act as one of the elimination signal for mitophagy [15]. These data suggest that oxidation of CL (and, perhaps, other lipids) is one of the early events that lead, via mitochondrial fragmentation and mitophagy, to myogenesis. This suggestion is in good agreement with the findings that high doses of mitochondria-targeted antioxidants inhibited myogenesis via inhibition of mitochondrial fragmentation and mitophagy [52–54]. Interestingly, mild depletion of mitochondrial ROS did not block in vitro fusion of primary myoblasts and even stimulated differentiation of myoblasts with some genetic defects [55]. Myogenesis is not the only example of the differentiation program that depends on mitoROS. In mesenchymal stem cells, mitoROS were found not only to initiate differentiation but also to contribute to cell fate determination [56]. Mitochondrial ROS (at least partially via modulation of mitochondrial dynamics and mitophagy) contribute also to keratinocyte differentiation within the epidermis and hair follicle development [57] and differentiation of adipocytes [58] and of immune cells [59]. MitoCLox could be a valuable tool for studies of these differentiation programs.

## 5. Conclusions

Here, we showed that molecules of a new mitochondria-targeted probe MitoCLox accumulated in mitochondria of living cells and reported the oxidation of mitochondrial lipids under conditions of oxidative stress. Ratiometric measurements of MitoCLox oxidation using flow cytometry revealed a very good dynamic range of the probe. Mitochondria-targeted antioxidant SkQ1 inhibited MitoCLox oxidation that was induced either by hydrogen peroxide or by organic hydroperoxide. The earlier findings demonstrated that CL (a) was selectively oxidized under conditions compatible to those of our experiments and (b) could be protected against oxidation by cationic mitochondria-targeted antioxidants [44–46]. We suggest that MitoCLox most likely preferably reports on the oxidation of CL, in agreement with results of molecular dynamic modeling that predicted a particular affinity of MitoCLox to CL [29]. Specifically, the application of MitoCLox revealed that the oxidation of lipids took place immediately after addition of hydrogen peroxide and preceded the drop in MMP. In the in vitro model of myogenesis, the use of MitoCLox revealed a cell subpopulation with an increased level of lipid oxidation and fragmented mitochondria. These cells were observed only after prolonged culturing of a dense culture of myoblasts, which is a necessary condition for the onset of myogenic differentiation. In sum, the new probe has demonstrated a notable potential for mitochondrial lipid peroxidation studies in living cells.

## Data Availability

The data used to support the findings of this study are included within the article.
